# Detection of Urothelial Bladder Cancer Based on Urine and Tissue Telomerase Activity Measured by Novel RT-TRAP-2PCR Method

**DOI:** 10.3390/jcm10051055

**Published:** 2021-03-04

**Authors:** Alexander Glukhov, Natalya Potoldykova, Mark Taratkin, Sergey Gordeev, Konstantin Polyakovsky, Ekaterina Laukhtina, Marco Moschini, Mohammad Abufaraj, Shahrokh F. Shariat, Marina Sekacheva, Dmitry Enikeev, Petr Glybochko

**Affiliations:** 1 Department of Biochemistry, Sechenov University, 119146 Moscow, Russia; urologystatement1@yandex.ru; 2 Faculty of Biology, Moscow State University, 119192 Moscow, Russia; urologystatement1@ya.ru; 3 Institute for Urology and Reproductive Health, Sechenov University, 119435 Moscow, Russia; potoldykovanv@gmail.com (N.P.); marktaratkin@gmail.com (M.T.); urologystatement1@yandex.by (K.P.); katyalaukhtina@gmail.com (E.L.); shahrokh.shariat@meduniwien.ac.at (S.F.S.); urologystatement1@yandex.ua (P.G.); 4 Department of Urology, Medical University of Vienna, 1090 Vienna, Austria; dr.abufaraj@gmail.com; 5 Department of Urology, Luzerner Kantonsspital, 6000 Lucerne, Switzerland; marco.moschini87@gmail.com; 6 Department of Urology and Division of Experimental Oncology, Urological Research Institute, Vita-Salute San Raffaele University, 20132 Milano, Italy; 7 Department of Urology, The University of Jordan, 11942 Amman, Jordan; 8 Computational Oncology Group, Sechenov University, 119435 Moscow, Russia; urologystatement1@yandex.com

**Keywords:** bladder cancer, non-invasive diagnosis of bladder cancer, urine biomarker, RT-TRAP-2PCR, non-isotopic TRAP assay

## Abstract

Purpose: To assess the diagnostic performance of urine telomerase activity (TA) in detecting bladder cancer (BCa) using the modified Telomeric Repeat Amplification Protocol (TRAP) and the Real Time Telomeric Repeat Amplification Protocol with double Polymerase Chain Reaction (RT-TRAP-2PCR). Methods: In this case-control study, matching urine (in the pre- and post-surgical period) and tissue samples from 68 patients with BCa were assessed for TA. As a control, 45 urine samples were examined from non-BCa patients. TA levels were measured using TRAP and RT-TRAP-2 PCR methods. Results: Preoperative urinary TA was elevated in 64 (94.1%) of the 68 BCa patients. Urine TA was undetectable in 44 control patients, while TA was detected in one patient with histologically verified cystitis. Sensitivity for BCa detection of 94.1% and specificity of 97.8% were observed for urinary TA, while tissue TA had 100% sensitivity and 97.8% specificity. Both urine and tissue TA levels were not significantly higher in patients with muscle-invasive disease compared to those with non-muscle invasive BCa (*p* > 0.05). Urine and tissue TA levels were not associated with higher tumor grade, stage, and number of tumors (*p* > 0.05). However, the association was found between higher urinary and tissue TA levels with tumor size ≥ 3 cm (*p* = 0.02 and *p* = 0.01, respectively). During the first postoperative year, 17 BCa patients experienced disease recurrence, and urinary TA was present in 14 (82.4%) of these patients. The sensitivity and specificity of urinary TA levels for BCa recurrence in patients with non-muscle invasive bladder cancer (NMIBC) during follow-up were 82% and 94.4%, respectively. Conclusions: This pilot study demonstrates a high diagnostic performance of urinary and tissue TA levels measured by a new RT-TRAP-2PCR method for detecting and monitoring BCa. Additionally, the association was found between higher urinary and tissue TA levels with tumor size ≥ 3 cm; however, higher TA levels failed for significant correlation with advanced tumor stage and grade. Our study could serve as a benchmark for the evaluation of novel biomarkers using the RT-TRAP-2PCR method.

## 1. Introduction

Despite extensive research efforts, there are no convincing data to support a reproducible clinical benefit of adding urinary biomarkers beyond cytology into the routine diagnosis of bladder cancer (BCa), its follow-up, and the decision-making process [1,2].

Telomerase has been shown to have diagnostic and prognostic value in various malignancies [3,4]. External telomerase activity (TA) stimulation does not appear to cause oncogenic transformation in normal cells, and isolated expression of the telomerase gene leads to cellular immortality [5]. Telomerase activity levels assessed with telomeric repeat amplification protocol (TRAP) seem to be good candidates for early detection of BCa. TA measured with TRAP was previously shown to be a promising biomarker with high sensitivity and specificity for BCa diagnosis [6,7]. However, the TRAP method is laborious and does not provide precise quantitative information. The currently used methods for telomerase activity identification based on a real time polymerase chain reaction (PCR) are a modification of the classical TRAP method, involving fluorescent dyes to visualize reaction products, for example, SYBR Green I or SYBR Gold, fluorescent-labeled primers, luminescent-labeled primers, radioactive-isotope labeled primers, and biotinylated primers [8,9,10,11]. These approaches require a large number of PCR cycles during one stage significantly changing the dependence between the rate of amplicon synthesis and the reaction time resulting in nonspecific products synthesis. These methods have a high cycle threshold significantly affecting the analysis quality. Moreover, their sensitivity is low if the sample lysate contains the reaction inhibitors and/or <190 cells [12]. 

We have developed a new real-time double telomeric repeat amplification (RT-TRAP-2PCR) technique. The proposed RT-TRAP-2PCR method is a modification of the classical method for telomerase activity identification by the TRAP method [13]. A key feature of the proposed approach is the second sequential real-time PCR reaction (PCR No. 2) with a different couple of primers. At stage II of PCR, we measure the total content of telomeric repeats newly synthesized at stage I of PCR with primers, one of which is a telomerizing oligonucleotide, and the other is a primer complementary to telomeric repeats in the genome of the analyzed biological object in the sample, that is, in fact, the analysis of the total content of all telomeric repeats (TTAGGG)n for vertebrates, including humans. This step enables a significant increase in the method sensitivity and specificity. To quantify telomerase activity in a sample, a calibration curve technique can be used involving an extract of a tumor cell line (with a known content of telomerase molecules per cell) or a double-stranded synthetic matrix ds(GGGTTA/CCCAAT)n as a positive control.

This study aimed to investigate the feasibility of the relative TA level assessment using the novel RT-TRAP-2PCR method in the urine and tissue samples obtained from BCa patients.

## 2. Experimental Section

We conducted a prospective study to identify TA level using the TRAP and RT-TRAP-2PCR in the urine and tissue samples obtained from BCa patients compared to controls. Patient samples were obtained between 2014 and 2017. This study was approved by the Ethics Committee at the I.M Sechenov First Moscow State Medical University, Moscow, Russia (Approval Protocol N° 02-14, 19 February 2014). Informed consent was obtained from eligible patients.

Urine samples from the second micturition of the day were obtained in order to be examined before and after the planned intervention to assess urinary cells for TA. We examined urine samples from the second micturition of the day because morning urine is not suitable for research due to possible cytolysis, as well as the presence of a large number of telomerase inhibitors contained in the morning urine. A small portion of the tumor tissue (approximately 2 mm) was, at the time of transurethral resection of bladder tumor (TURBT), immediately placed in an impermeable container, quick-frozen in liquid nitrogen, and stored at −70 °C. Immediate and/or adjuvant intravesical therapy was administered at the discretion of the treating physician. 

In patients with non-muscle invasive bladder cancer (NMIBC), the frequency of cystoscopy and imaging studies were performed based on stage and grade in accordance with current EAU guidelines [12]. The follow-up period for all BCa patients was one year. TA was analyzed in the urine cell material every three months after the initial resection (at three, six, nine, and twelve months). In the histologically confirmed muscle-invasive bladder cancer (MIBC) cases, radical cystectomy was performed in a timely fashion. 

Similar to the cases, urine samples from the second micturition of the day were examined among all participants in the control group. TA was assessed in non-cancerous appearing bladder tissue obtained during a diagnostic cystoscopy performed for other reasons than cancer detection. TA was also determined in 19 biopsies of the bladder wall from 20 patients with chronic cystitis obtained after cystoscopy with biopsy, the performance of which was carried out only for clinical indications (focus of hyperemia, area suspicious for leukoplakia, cystic cystitis, presence of bullous edema). Due to the fact that the exact association between inflammation and TA is currently unclear [13], we decided to analyze the dependence of the relative level of TA in urine and tissue samples on the presence of leukocyturia, in order to minimize the possibility that inflammation would be a confounding factor in our study. All patients were clinically and histologically confirmed to be cancer-free. None of the control patients had a history of urothelial carcinoma. 

### 2.1. Methods

The biological samples were washed in a sterile phosphate buffered saline (PBS) solution and immediately frozen. Samples were stored at −70 °C. Before preparing the lysates, the samples were thawed in ice. Then a cold CHAPS lysis buffer was added containing 10 mM of Tris-HCl (pH 7.5), 1 mM of MgCl_2_, 1 mM of EGTA, 0.1 mM of PMSF, 5 mM of β-mercaptoethanol, 0.5% (*w*/*v*) of CHAPS and 10% (*w*/*v*) of glycerol. 1 μL of CHAPS buffer was added per 1000 cells. The sample was then resuspended in the buffer and incubated on ice for 30 min. During incubation, the sample was vigorously stirred every 5 min on a Vortex apparatus for several seconds. The obtained cell extract was centrifuged (ELMI CM-6MT centrifuge, rotor 6M, Latvia) at 12,000× *g* for 30 min at 4 °C. Without touching the precipitate, the supernatant was carefully transferred in 25 μL portions into clean microtubes and quickly frozen in liquid nitrogen. The extracts were stored at −70 °C. 

Protein concentration in cell and tissue extracts was identified with Coomassie Brilliant Blue G-250 dye following the Bradford assay [14]. The oligonucleotide substrate elongation and subsequent amplification was carried out in 50 μL of a reaction mixture containing 67 mM of Tris-HCl (pH 8.8); 16.6 mM of (NH_4_)_2_SO_4_; 0.01% of Tween-20; 1.5 mM of MgCl_2_; 1 mM of EGTA; 50 mM of each dNTPs; 0.1 µg of TS-primer (5′-ATTCCGTCGAGCAGAGTT-3′). For a positive control, 1 μL of a cell extract of the tumor cell line K562 (chronic promyeloid leukemia) diluted with CHAPS lysis buffer and equivalent to 100 tumor cells with a protein concentration of 0.04 μg/μL was used. The method sensitivity analysis involved an extract containing 100 cells in 1 μL diluted with lysis buffer CHAPS to final values of 10, 40, and 80 cells per 1 μL. For a negative control, 1 μL of CHAPS buffer was used instead of the extract. Telomerase-mediated extension of the TS primer resulted from the reaction mixture incubation at 37 °C for 25 min. At the end of the incubation, the mixture was incubated for 5 min at 94 °C to inactivate telomerase. Then, 0.1 μg of CX primer (5′-CCCTTACCCTTACCCTTACCCTAA-3′) and 2.5 units SmarTaq DNA Polymerase were added to each sample (TRAP-PCR reaction No.1 (PCR No.1)). The reaction mixture was amplified for 35 PCR cycles in the following mode: 94 °C–60 s, 50 °C–60 s, 72 °C–90 s (TRAP-PCR reaction No.2 (PCR No.2)). Then, 25 μL of an incubation mixture was prepared, containing 67 mM Tris-HCl (pH 8.8); 16.6 mM (NH_4_)_2_SO_4_; 0.01% of Tween-20; 1.5 mM of MgCl_2_; 50 mM of each dNTPs; 0.9 μM of TelG primer (5′-ACACTAAGGTTTGGGTTTGGGTTTGGGTTTGGGTTAGTGT-3′) and TelC primer (5′-TGTTA GGTATCCCTATCCCTATCCCTATCCCTATCCCTAACA-3′), as well as 0.75 × SYBR Green I (dye), 1 × ROX (reference dye), 1 M of betaine, and 1.25 units SmarTaq DNA Polymerase. Then, 1 μL of reaction mixture after PCR reaction No. 1 was added to the prepared mixture and 22 cycles of real time PCR were performed in the following mode: 95 °C–15 min; 2 cycles in the mode: 94 °C–15 s, 49 °C–15 s; 20 cycles in the mode: 94 °C–15 s, 62 °C–10 s, 72 °C–30 s with signal detection. 

The TA level was determined in clinical samples relative to the telomerase-positive control k562 cells lysate using a comparative 2ΔCt method. To separate false negative results from true negative ones and to identify telomerase activity inhibitors, inhibitory analysis of the protein extract was required. All tests were done in duplicate.

Protein concentration was measured by means of a micro-biuret method in each lysate obtained from urine cell sediment from patients and controls. The same amount of protein lysates was added to the reaction mixture from both bladder cancer patients and the controls to analyze telomerase activity. Thus, we performed protein normalization of cell lysates from the urine of patients.

For a semi-quantitative TA evaluation, the ImageJ graphic editor (NIH) was used to compare the staining intensity of strips corresponding to telomerase reaction products, urine cell material, and tissue extract studied with that of the control sample strips. The control staining intensity was taken for one unit of normalized telomerase activity (NTA). When three or more one 6 bp long telomere repetitions expressed as horizontal stripes were identified in TRAP products, the sample under study was considered telomerase-positive and subject for calculation. All tests were done in duplicate.

The ImageJ program was used to process images in a gel of discrete bands of TRAP products in a positive control sample (telomerase-positive cell line) and compare them with symmetrical bands of TRAP products in the samples under study. The unit (100%) was defined as the intensity of the luminescence of specific bands of the positive control sample. All the samples under study were normalized to this luminescence intensity. In this case, the luminescence intensity was analyzed starting from the second lower band of the TRAP product. The lowest first fragment in the gel corresponding to the first specific fragment of the TRAP product and the nonspecific PCR duplex of CX and TS primers was not taken into account in the calculation of the luminescence intensity.

### 2.2. Statistical Analysis

The study endpoints were sensitivity (the proportion of cancer patients who were correctly identified by the test or procedures), specificity (the proportion of healthy individuals who were correctly identified), negative (NPV), and positive predictive values (PPV). The continuous data were presented as medians and quartiles (IQR). Differences between variables were tested using the Kruskal–Wallis, Mann–Whitney, Chi-Square, and Spearman correlation as appropriate. Pearson’s chi-square test was used to realize intergroup comparison. The results were considered significant at *p* < 0.05. All tests were two-sided. Statistical analyses were performed using SPSS 21 (IBM Corp, 2012, Armonk, NY, USA). 

## 3. Results

Overall, 113 participants were included in our study. Among them, 82 (72.5%) participants were men and 31 (27.5%) were women. Patient characteristics are shown in Table 1. The cases included 68 patients with primary BCa, whose urine pellets were assessed prior to and after surgery, and their matching tumor specimens were assessed for TA activity. Postoperative bleeding did not require bladder irrigation with saline for 24 h postoperatively. Thirty-nine patients received single, immediate, postoperative intravesical instillation of mitomycin C and four patients received Bacillus Calmette-Guerin (BCG) therapy. The control group included 45 patients with chronic cystitis, kidney cysts, or prostate hyperplasia. Median age was significantly higher for BCa patients (64.5 years) compared to the controls (54.4 years, *p* = 0.04). Urine cell material from all these participants and bladder tissue samples from 19 of these patients with chronic cystitis were obtained.

### 3.1. Measurement of Urine and Tissue Specimens TA

In the first phase of the study, we used the classic non-isotope TRAP method allowing the analysis of the relative TA level in the samples expressed in the NTA regarding a positive control. In the case of the urine cell material lysates, the samples were found with low relative TA levels undetectable even by 28 PCR cycles in TRAP analysis. With the increase of PCR cycles number to 35, it was possible to detect the TA. However, non-specific TRAP products appeared in the negative control sample because of the amplification of the TS and CX primers dimer. We introduced a new more sensitive TA analysis: RT-TRAP-2PCR.

**Figure 1:**
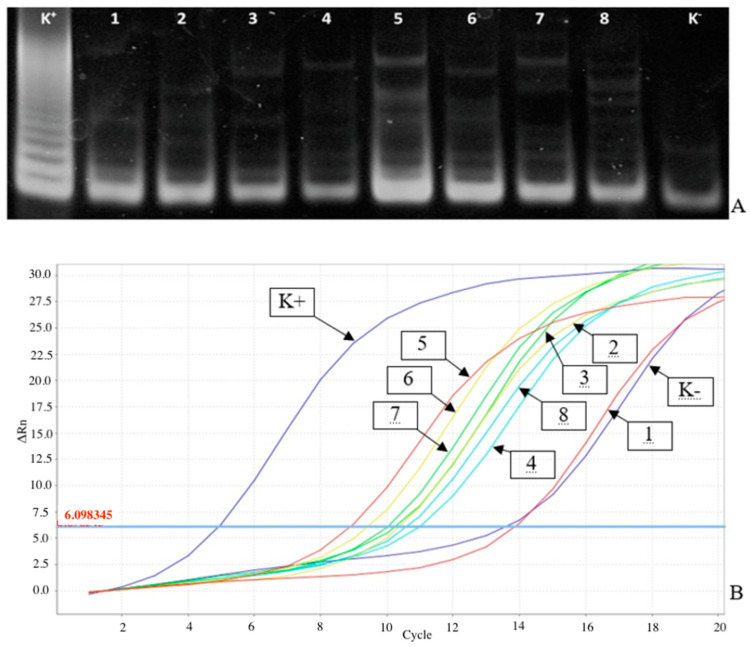
Telomerase activity (TA) analysis in tumor tissue and urine cell material samples from a patient with bladder cancer. TA analysis in a tumor tissue (tracks 1–4, A, and curves 1–4, B) and urine cell material (tracks 5–8, A, and curves 5–8, B) samples from a patient with bladder cancer. Reaction samples contained various amounts of lysate protein: 8 μg (tracks 1, 5, A, and curves 1, 5, B), 4 μg (tracks 2, 6, A, and curves 2, 6, B), 2 μg (tracks 3, 7, A, and curves 3, 7, B), and 0.7 μg (tracks 4, 8, A, and curves 4, 8, B). Track K− is a negative control sample (telomeric repeat amplification protocol (TRAP) in the absence of protein extract). Track K+ is a positive control (0.4 μg of the K562 cell line extract). (**A**) Electrophoresis of TRAP-analysis products for TA detection. 10% PAAG, dyed with SYBR GOLD, UV photography. (**B**) Analysis of telomerase activity by RT-TRAP-2PCR. For the reaction, 1.25 μL of reaction mixture was taken in TRAP.

As Figure 1A shows, it was difficult to document the gel to evaluate TA in the study of tumor tissue and urine cell material samples with relatively low TA contents. This fuzzy TRAP products pattern may result, for example, from the presence of TA inhibitors in the samples or low telomerase-positive cell contents. In the study of these samples by the RT-TRAP-2PCR, it is possible to find curves with significant Ct values to calculate the NTA values relative to positive and negative control samples by the 2ΔCt criterion (Figure 1B). 

### 3.2. TA Levels in Urine and Tissue of Cases and Controls

Among sixty-eight patients with histologically confirmed BCa, sixty-four (94%) patients had telomerase-positive expression in their preoperative urine pellets. TA expression was no longer measurable in the urine pellets obtained (approximately 24 h after TURBT) at the first postoperative urine collection. TA was absent in 44 (97.7%) of 45 urine pellets of the control group. The only false-positive test was in a female patient with histologically verified cystitis. Urinary TA level for that patient was 1.0 units of NTA, while tissue TA level was 2.19 units of NTA. TA was more expressed in BCa patients compared to controls (*p* < 0.001), with a sensitivity for BCa detection of 94.1%, a specificity of 97.8%, PPV of 98.4%, and NPV of 91.6%.

### 3.3. TA Expression in Tissue Samples

**Figure 2:**
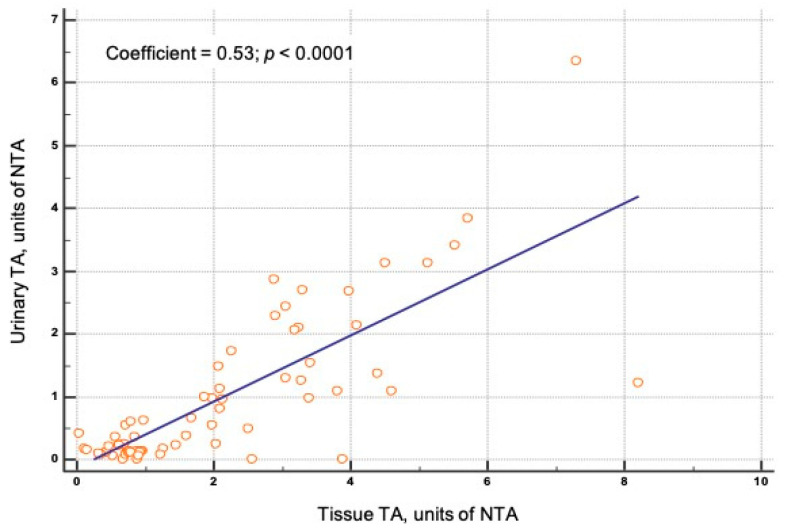
Plot showing pearson correlation analyses between urinary and tissue Telomerase activity (TA) levels. The correlation coefficient is displayed as an abline in the plots. NTA: normalized telomerase activity.

Sixty-eight samples of tumor tissue from the BCa group were examined for TA expression; TA was identified in all these cases. The control group included 19 bladder tissue samples from patients with cystitis; TA was measurable in one sample. TA levels were expressed more in the tumor tissue samples of BCa patients compared to that of controls (*p* < 0.001). Sensitivity of TA measured in tissue for BCa detection was 100%, specificity 97.8%, PPV 98.5%, and NPV 100%. Additionally, higher urinary TA levels were correlated with a higher TA expression in BCa tissue samples (Figure 2).

### 3.4. Association of Urine and Tissue TA Levels with Clinicopathologic Features

**Figure 3:**
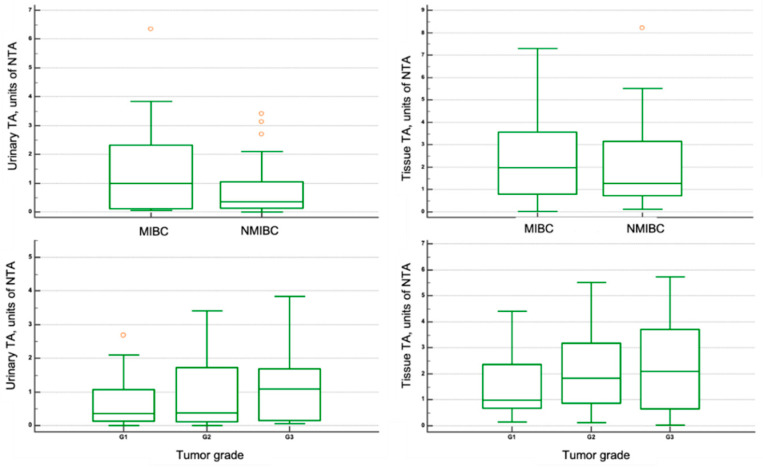
Boxplots showing the urinary and tissue Telomerase activity (TA) levels in BCa patients with different tumor stages and grades. NTA: normalized telomerase activity; NMIBC, non-muscle-invasive bladder cancer; MIBC, muscle-invasive bladder cancer.

Among 68 BCa patients, 43 patients had NMIBC (63.2%), and 25 patients had MIBC (36.8%). Although an increase in both urine and tissue TA levels was observed from NMIBC to MIBC, there were no statistically significant differences (*p* = 0.12 and *p* = 0.42, respectively) (Figure 3, Table 2). Twenty-five BCa patients had grade 1 (36.8%), 28 patients—grade 2 (41.2%), and 15 patients—grade 3 (22%). Despite an increase in both urine and tissue TA levels observed from histologic grades 1 to 3, it did not reach statistical significance (all *p* > 0.05). No association was found between urinary and tissue TA levels with tumor stage or number of tumors (all *p* > 0.05). While the significant association was found between higher urinary and tissue TA levels with tumor size ≥ 3 cm (*p* = 0.02 and *p* = 0.01, respectively). 

Among the controls, 14 patients had BPH (31.1%), 20 patients—chronic cystitis (44.4%), and 11 patients—renal cysts (24.4%). We analyzed the dependence of the relative level of TA in urine samples and tissue samples on the presence of leukocyturia in the main and control groups, and we failed to find a statistically significant association between TA and leukocyturia. There was no association of either urine or tissue levels of TA with leukocyturia (*p* = 0.25 and *p* = 0.89, respectively) as well as erythrocyturia (*p* = 0.5 and *p* = 0.9, respectively) in the control group. In BCa patients, we also did not find an association of urine or tissue levels of TA with leukocyturia (*p* = 0.16 and *p* = 0.2, respectively). Our analyses indicated that there was no relationship between telomerase activity in samples and erythrocyturia in the main group: there was no statistically significant difference in telomerase activity in urine cell sediment with or without erythrocyturia (*p* = 0.5).

### 3.5. Urine TA Levels during Follow-Up of Patients with NMIBC

During one-year follow-up, BCa recurrences were detected in 17 patients with NMIBC. TA was detected in the urine of 14 (82.4%) patients. In patients with BCa recurrence, the relative TA level in the urine cell material was 0.98 units of NTA (*p* < 0.05), and no units were detected in those without BCa recurrence. A comparative analysis of TA from the main group in samples of urine cell material and tumor tissue showed a significant predominance of relative TA level in tumor tissue samples till 2.11 units of NTA, compared to that in the tumor tissue urine cell material 0.97 units of NTA (*p* < 0.001). The sensitivity, specificity, PPV, and NPV of urinary TA levels for BCa recurrence in patients with NMIBC during follow-up were 82%, 94.4%, 100%, and 94.4%, respectively. 

## 4. Discussion

In the present pilot study, we developed the novel RT-TRAP-2PCR technique. Urine TA measured by RT-TRAP-2PCR method seems to have high performance in detecting BCa at initial diagnosis as well as during follow-up given its high sensitivity, specificity, PPV, and NPV for BCa in both settings. We found a high concordance of TA expression between the urine and BCa tissue samples. 

TRAP and RT/PCR are used to detect TA and analyze its catalytic subunit gene (hTERT) expression in urine samples to diagnose BCa [15,16]. However, high false-positive rates were detected in patients with urinary tract infections and/or inflammatory bladder conditions. When we measured TA by non-isotope modification of TRAP followed by TRAP products analysis by electrophoresis, we encountered a problem with the TA detection using low-protein lysates. Therefore, and to increase the sensitivity of the analysis, we increased the number of PCR cycles that caused an increase in the false-positive results associated with the synthesis of non-specific TRAP products. We, therefore, utilized the new RT-TRAP-2PCR method, which is based on an additional stage of PCR in the non-isotope TRAP method [17].

The tissue TA has been widely studied in different urological malignancies. For example, the sensitivity and specificity of tissue TA in patients with prostate cancer were 70–94% and 88–99%, respectively [16,18]. In the context of BCa, it was previously reported that the urine TA sensitivity and specificity for BCa diagnosis ranged between 61–93% and 42–88%, respectively [19]. Compared to previous studies, we found a higher diagnostic performance of urinary TA measured using RT-TRAP-2PCR with a sensitivity of 94% and a specificity of 98%, while tissue TA expression had 100% sensitivity and 98% specificity. Additionally, we found a significant association between higher urinary and tissue TA levels with tumor size ≥3 cm; however, higher TA levels failed for significant correlation with advanced tumor stage and grade. In agreement with our results, Casadio et al. reported that patient age, tumor grade, or stage at diagnosis were not associated with TA [19]. In contrast, other investigators found that clinical stage or grade can affect the TA detection accuracy [20]. These controversial results could be explained by a high heterogeneity of BCa. We believe that TA level assessment using the novel RT-TRAP-2PCR method in tissue samples should be studied in the context of different molecular subtypes and variants of histology.

Our findings suggest that TA is also unlikely to miss patients at risk of BCa or those who experience disease recurrence during follow-up. Patients with BCa will benefit from a sensitive non-invasive biomarker, especially in the follow-up settings where physicians can avoid regular urethrocystoscopy [21]. TA alone or in combination with other biomarkers can play a role in the early detection of recurrence, especially in patients at higher risk of developing BCa [22]. Such findings shall also pave the way for future efforts to assess the possible role of telomerase-directed therapies in BCa. 

Most of the currently available biomarkers are not assessed in routine urologic practice. It is costly and time-consuming, among other reasons. Telomerase detected by the new RT-TRAP-2PCR method is not an exception. Our technique includes one more PCR stage, which increases the analysis time by 1.5 h compared to the prototype. However, it makes RT-TRAP-2PCR highly sensitive to telomerase activity compared to the standard RT-TRAP-PCR method. It is known that urine contains a large number of various metabolites and transformation products of xenobiotics, among which may be inhibitors of DNA polymerases. This significantly reduces the sensitivity of the PCR assay and leads to false-negative results. RT-TRAP-2PCR makes it possible to mitigate the negative effect of inhibitors to a certain degree and avoid false-negative results, and repeat tests. Additionally, the cost of the novel method is not significantly higher than that of the prototype. Nevertheless, for a biomarker to add value and impact clinical decision making, the tests need to be faster and cheaper.

Our study is not devoid of limitations. The main limitation of the study was the small cohort size, which may have limited power to find statistically and/or clinically significant associations. However, among the strength of our study, we would like to highlight its prospective design. Hence, we are unable to significantly increase our study population. Nevertheless, we believe that this number of patients is appropriate for the pilot study developing a new RT-TRAP-2PCR method. Second, we did not account for the potential effect of external factors resulting in vulnerability and inactivation of the enzyme. Third, TA, inflammation and carcinogenesis have an unclear association, resulting in a disadvantage of the study, because patients with chronic cystitis were included in the control group. However, TA was almost undetectable in the control group. Moreover, our analyses failed to find the association between TA and leukocyturia in both groups. For this reason, we decided that patients with chronic cystitis may not be excluded from the study. Nevertheless, to validate our results, further in-depth large-scale studies are needed. 

## 5. Conclusions

This pilot study demonstrates a high diagnostic performance of urinary and tissue TA levels measured by RT-TRAP-2PCR for detecting and monitoring BCa. Additionally, the association was found between higher urinary and tissue TA levels with tumor size ≥ 3 cm; however, higher TA levels failed for significant correlation with advanced tumor stage and grade. Further well-designed large-scale studies are needed to validate our findings. Our study could serve as a benchmark for the evaluation of novel biomarkers using the RT-TRAP-2PCR method.

## Figures and Tables

**Table 1:** Clinicopathologic characteristics of 68 patients with bladder cancer (cases) and 45 controls as well as its association with TA levels in urine and tissue samples.

Characteristic	Cases (*n* = 68)	Controls (*n* = 45)
Age at diagnosis (years), median (range)	64.5 (29–85)	54.4 (21–80)
Gender, *n* (%)		
Female	11 (16.2%)	20 (44.4%)
Male	57 (83.8%)	25 (55.6%)
Tumor stage, *n* (%)		–
NMIBC	43 (63.2%)	
MIBC	25 (36.8%)	
pT Stage, *n* (%)		
T1	42 (62%)	
T2	15 (23%)	
T3a	4 (6%)	–
T3b	5 (7%)	
T4	1 (2%)	
Histological grade, *n* (%)		
G1	25 (36.8%)	
G2	28 (41.2%)	–
G3	15 (22%)	
Tumor size, *n* (%)		
<3 cm	42 (61.8%)	–
≥3 cm	26 (38.2%)	
Number of tumors, *n* (%)		
Single	38 (55.9%)	–
Multiple	30 (44.1%)	
Benign disease, *n* (%)		
BPH		14 (31.2%)
Chronic cystitis	–	20 (44.4%)
Renal cysts		11 (24.4%)

Abbreviations: NMIBC, non-muscle-invasive bladder cancer; MIBC, muscle-invasive bladder cancer; G, grade; BPH, benign prostatic hyperplasia. T3a (microscopically) and T3b (macroscopically, extravesical mass)–stages according to EAU guidelines.

**Table 2:** Relationship between Telomerase activity (TA) level and clinicopathologic characteristics.

Characteristic	Urinary TA	Tissue TA
Tumor stage		
NMIBC	0.35 (0.13; 1.1)	1.27 (0.72; 3.16)
MIBC	1.0 (0.12; 2.32)	1.98 (0.79; 3.56)
*p* value	0.12	0.42
Histological grade		
G1	0.35 (0.14; 1.07)	0.98 (0.67; 2.35)
G2	0.37 (0.12; 1.72)	1.83 (0.87; 3.18)
G3	1.08 (0.15; 1.68)	2.10 (0.66; 3.71)
*p* value	0.51	0.26
Tumor size		
<3 cm	0.24 (0.11; 0.95)	0.94 (0.69; 2.13)
≥3 cm	1.09 (0.23; 2.14)	2.70 (0.97; 3.98)
*p* value	0.02	0.01

TA level in units of normalized telomerase activity (NTA), median (IQR, interquartile range). NMIBC, non-muscle-invasive bladder cancer; MIBC, muscle-invasive bladder cancer; G, grade.

## Data Availability

The data presented in this study are available on request from the corresponding author. The data are not publicly available due to their containing information that could compromise the privacy of research participants.
